# Rat Sarcoma Virus Family Genes in Acute Myeloid Leukemia: Pathogenetic and Clinical Implications

**DOI:** 10.3390/biomedicines13010202

**Published:** 2025-01-15

**Authors:** Shaimaa Khattab, Adriatik Berisha, Natalia Baran, Pier Paolo Piccaluga

**Affiliations:** 1 Biobank of Research, IRCCS Azienda Ospedaliera, Universitaria di Bologna, Policlinico di S. Orsola, 40138 Bologna, Italy; shaymaakhattab@hotmail.com; 2 Department of Medical and Surgical Sciences, Bologna University School of Medicine, 40138 Bologna, Italy; 3 Medical Research Institute, Alexandria University, Alexandria 21526, Egypt; 4 Division of Hematology, University of Pristina, 10000 Pristina, Kosovo; 5 School of Medicine, University of Zagreb, 10000 Zagreb, Croatia; 6 Department of Leukemia, The University of Texas MD Anderson Cancer Center, Houston, TX 77030, USA; 7 Section of Experimental Hematology, Institute of Hematology and Transfusion Medicine, 02-776 Warsaw, Poland

**Keywords:** RAS, oncogene, AML, chemoresistance, RAF-MEK-ERK1/2, RAS-like proteins

## Abstract

Acute myeloid leukemias (AMLs) comprise a group of genetically heterogeneous hematological malignancies that result in the abnormal growth of leukemic cells and halt the maturation process of normal hematopoietic stem cells. Despite using molecular and cytogenetic risk classification to guide treatment decisions, most AML patients survive for less than five years. A deeper comprehension of the disease’s biology and the use of new, targeted therapy approaches could potentially increase cure rates. *RAS* oncogene mutations are common in AML patients, being observed in about 15–20% of AML cases. Despite extensive efforts to find targeted therapy for *RAS*-mutated AMLs, no effective and tolerable RAS inhibitor has received approval for use against AMLs. The frequency of *RAS* mutations increases in the context of AMLs’ chemoresistance; thus, novel anti-RAS strategies to overcome drug resistance and improve patients’ therapy responses and overall survival are the need of the hour. In this article, we aim to update the current knowledge on the role of RAS mutations and anti-RAS strategies in AML treatments.

## 1. Introduction

Acute myeloid leukemia (AML) is a highly aggressive clonal blood malignancy that arises from the malignant transformation of a single hematopoietic cell [1]. The hallmarks of AML are maturation arrest, uncontrolled proliferation of blast cells in the bone marrow and peripheral blood, and frequent extramedullary spread to any other organs, such as the spleen, lymph nodes, lungs, liver, or meninges [2]. The clonal blast cell population in AML grows uncontrollably, resulting in a life-threatening condition. Despite significant advancements in diagnosis and treatment, AML continues to exhibit a five-year survival rate [3]. The disease affects both adults and children, but it is more prevalent in adults, particularly those aged 65 years or older [4].

**Figure 1:**
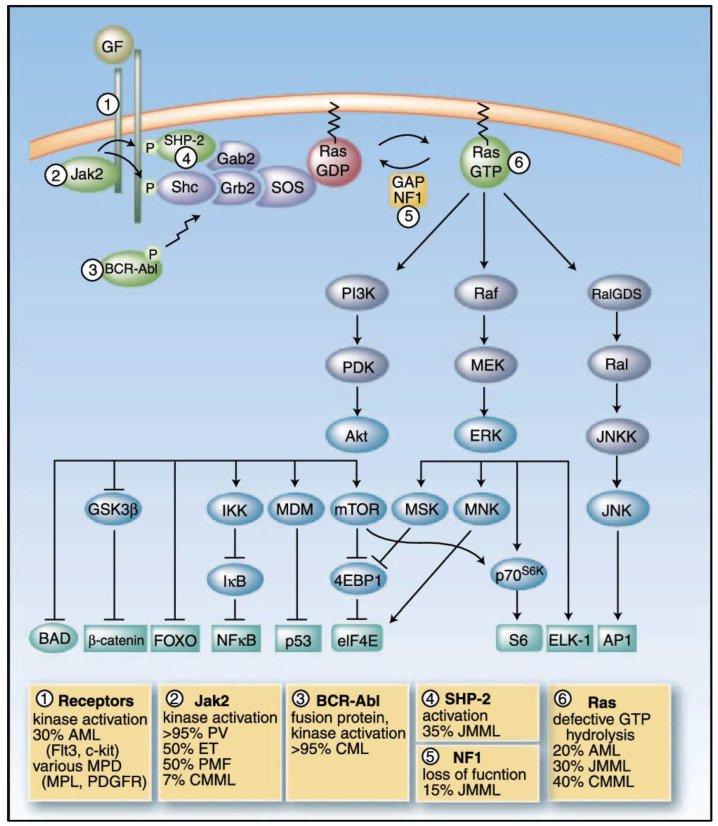
Schematic of Ras signaling and its involvement in myeloid malignancies. Ras proteins play a critical role in mediating intracellular signaling in response to extracellular growth factors by toggling between inactive GDP-bound and active GTP-bound states. Ras activation is initiated when growth factor receptors, upon ligand binding, assemble a complex involving adapter proteins, the phosphatase SHP-2, and guanine nucleotide exchange factors (GEFs) like SOS. These GEFs promote the exchange of GDP for GTP on Ras, leading to its activation. Once activated, Ras-GTP interacts with a variety of downstream signaling proteins, initiating a cascade of signaling events that regulate vital cellular processes such as gene expression (β-catenin, FOXO, NF-κB, p53, ELK1, AP1), translation (eIF4E, S6), and apoptosis (BAD). The Ras signaling pathway is tightly controlled through the hydrolysis of GTP to GDP, a process facilitated by GTPase-activating proteins (GAPs) like p120GAP and neurofibromin, which turn off Ras activity. Mutations in Ras or its regulators are implicated in various malignancies, including myeloid cancers, with specific mutations being noted. GF, growth factor; GEF, guanine nucleotide exchange factor; GAP, p120 GTPase-activating protein; NF1, neurofibromin; MPD, myeloproliferative disorders; PDGFR, platelet-derived growth factor receptor; CMML, chronic myelomonocytic leukemia; CML, chronic myelogenous leukemia; mTOR, mammalian target of rapamycin (adapted from [7]).

The *RAS* oncogene, the earliest activated proto-oncogene detected in human malignancies, has been found in 25% of human cancers [5], with a high prevalence in hematologic malignancies [6] (Figure 1).

Moreover, *RAS* mutations exhibit notable oncogenic properties and activate downstream signals [8,9]. Solid malignancies with these mutations show more aggressive phenotypical characteristics than those without a *RAS* mutation. Furthermore, RAS mutations frequently result in a more dismal prognosis and reduced overall survival rates for patients compared to wild-type *RAS* [10]. The activation of *RAS* oncogenes and the loss of tumor suppressor genes, such as *TP53*, contribute significantly to tumor formation and progression. Moreover, it is essential for additional genetic alterations to interact with mutant *RAS* cells to fully execute their transformation into malignant cells [8]. RAS proteins serve as crucial keys in cellular signaling. They skillfully combine information from external stimuli and activate cell surface receptors to effectively dictate the destiny of cells through a complex network of signal transduction pathways, namely the RAS-RAF-MEK-ERK and PI3K-AKT–mTORC pathways [11,12].

**Figure 2:**
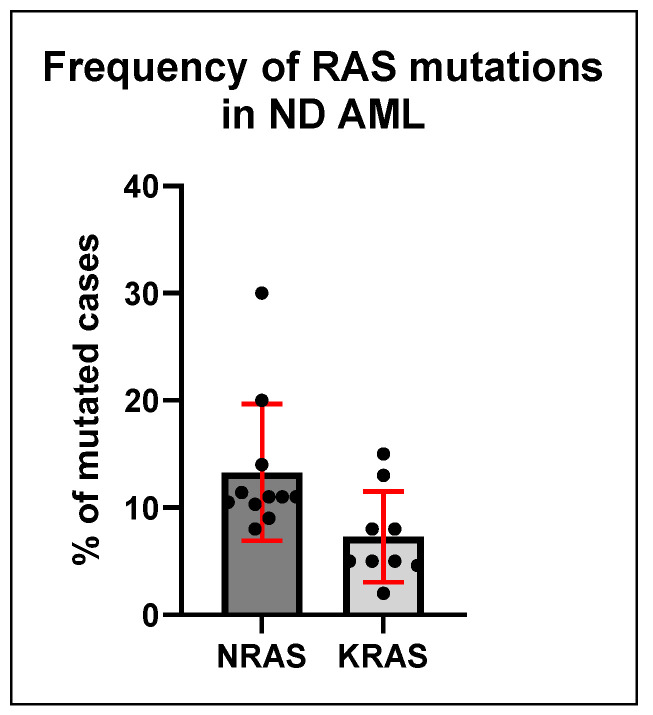
Prevalence of NRAS and KRAS mutations in AML. Current research indicates that NRAS mutations are significantly more prevalent in AML patients than KRAS mutations. NRAS mutations are found in approximately 11–12% of cases, whereas KRAS mutations are reported in about 5% of cases. The error bars represent the range of the highest and lowest prevalence rates reported in the literature [8,21,22,23,24,25].

AML undergoes frequent *RAS* oncogene mutations, as has been observed in 15–20% of AML cases [2]. *NRAS* mutations have been the most prevalent *RAS* mutation, being identified in 10–15% of AML patients, and *KRAS* mutations are found in 5–10% of patients [13] (Figure 2). A small number of AML cases with *HRAS* mutations have been reported [2]; however, it remains unclear if these mutations have any predictive importance for AML patients [14]. Recent research has shown some predictive value in regard to mutated *RAS* in newly diagnosed AML patients [2] and linked it to venetoclax resistance [2,15]. Despite decades of research, no potent *RAS* inhibitor has received approval for use against AML yet. This has led to the widespread belief that *RAS* oncoprotein is an “undruggable” cancer target [3]. Despite previous disappointments, recent developments in our understanding of RAS biological function, coupled with advanced methods and technologies for drug discovery, have brought us closer than ever to finding a drug that targets RAS [2]. In fact, there are several ongoing clinical trials of molecules that target the RAS or its downstream targets [16,17,18,19,20].

In this article, we review the basics of the *RAS* proto-oncogene, the prevalence and prognostic value of *RAS* mutations in AML, the therapeutic interventions targeting *RAS* mutation in AML, and the mechanisms of chemotherapeutic drug resistances in *RAS*-mutated AML. Since the field is continuously evolving, we aimed to provide a timely update and summarize the key information needed for readers to approach this subject. Our literature research was conducted using PubMed, EndNote, Web of Science (Clarivate), and Google Scholar, with “RAS” and “acute myeloid leukemia” (or “AML”) being used as keywords. We primarily considered original research articles and review articles published between 2014 and 2024.

## 2. Basic Structure of RAS Proteins

The RAS protein is a node protein in several signaling pathways that regulates both normal cell maturation and tumorigenesis [26]. The RAS oncoprotein family comprises three GTP (guanine nucleotide-binding) proteins, namely HRAS (Harvey RAS), KRAS (Kirsten RAS), and NRAS (neuroblastoma RAS). The KRAS protein has two types, K4A and K4B, both of which occur through alternative splicing [27,28,29]. The HRAS, KRAS, and NRAS proteins are widely expressed in diverse types of cells. KRAS is present in nearly every cell type. The *KRAS* gene is crucial for normal mouse development, and *KRAS* knockout was found to be lethal in mice due to severe anemia and severe hepatic injury [30]. On the other hand, *NRAS* and *HRAS* appear less crucial; *NRAS* and *HRAS* knockout mice [3,27,29] exhibited normal phenotypes and moderate immunodeficiency, respectively, indicating that these genes are not as widely expressed [31]. The reason for this variable expression could be the distinct chemical roles of the three proteins, as NRAS and HRAS show lower levels of essentiality than KRAS [3,27,29].

HRAS, KRAS, and NRAS have a high degree of sequence identity, especially in the G-domain, which is about 85% identical across the RAS family; this domain (aa 1-166), is responsible for binding guanine nucleotides (GDP and GTP) and is crucial for their GTPase activity. The sequence homology is particularly strong in the regions involved in nucleotide binding and hydrolysis, such as the P-loop, switch I, and switch II regions.

Several amino acids are critical in this context. In particular, G12, G13, and Q61 residues are frequently mutated in cancers and play a crucial role in GTPase activity. Mutations often lock RAS in an active GTP-bound state, promoting oncogenic signaling. The CAAX motif, which is the last four amino acids of the C-terminal, is the target of crucial post-translational modifications that modulate RAS shifts and binding to the cell membrane [32].

By contrast, the C-terminal Hypervariable Region (HVR) shows significant divergence, contributing to differences in subcellular localization and specific protein interactions. This region particularly of (aa 166–188/189) undergoes various post-translational modifications, such as palmitoylation in HRAS, the influence of which are essential to RAS binding to cell membranes [33].

### 2.1. Post-Translational Modification for RAS Activation

Some critical post-translational modifications are crucial to enhancing the hydrophobicity of RAS, leading to better membrane identification and anchoring. For example, farnesylation occurs at the CAAX motif of the four isoforms of RAS [34]. Additionally, palmitoylation occurs upstream of the CAAX motif in NRAS, HRAS, and KRAS4A but not in KRAS4B, as it does not have the palmitoylation site, so its membrane anchoring is mediated through other methods, such as farnesylation and a poly-basic stretch of lysine [35,36]. These post-translational protein modifications are essential for proper RAS biological function because they allow RAS conversion from an inactive cytoplasmic protein to a fully active membrane-associated protein.

### 2.2. Upstream RAS Activation and Deactivation

RAS becomes active in response to mitogens, cytokines, and growth factors [15,37,38,39]. The activation and inactivation of RAS are directly linked to the conformational changes that occur within the switch region. These changes have a significant impact on GEF (guanine nucleotide exchange factor) and GAP (GTPase-activating protein) binding: RAS activation is closely linked to GEF binding, and its inactivation is linked to GAP binding [40,41,42]. When inactive, RAS is restricted to the plasma membrane and attached to GDP [40,41,42]. After ligand interaction with its cell surface cognate receptor, which often has intrinsic or extrinsic tyrosine kinase activity, crucial tyrosines in the receptor’s cytoplasmic domain become phosphorylated, providing docking sites for the adaptor molecule GRB2 (growth factor receptor-bound protein 2) [43,44]. GRB2 enhances recruitment of GEFs for the minute G proteins of the RAS family [43,44]. GEFs become activated and bind to RAS. They interfere with the nucleotide binding in switch regions I and II, leading to the release of GDP and causing RAS to bind to GTP. Because of the higher levels of cellular GTP compared with GDP, RAS has more affinity to GTP. This process is regulated by the RAS guanine nucleotide exchange factor SOS (son of sevenless) [45]. There is another method of RAS upstream activation through growth factor receptor tyrosine kinases, including the IGF-1 (insulin-like growth factor 1) receptor via intermediate compounds such as insulin receptor substrate proteins that bind to GRB2 [28,29,43,46,47]. RAS has its own intrinsic GTPase hydrolyzing ability which is capable of stopping the signal in the absence of other extrinsic inhibitory regulators [29,32]. For instance, the arginine finger effectively stabilizes the transition state and increases GTPase hydrolysis by a thousand times [29,32].

### 2.3. The Downstream Action of RAS Protein Is the RAF-MEK-ERK1/2 Pathway

The activation of RAF is facilitated by a conformational shift that occurs when RAS-GTP binds to the RBD and cysteine-rich domains, disrupting 14-3-3 dimer binding [48,49]. This shift releases the catalytic domain, which is then primed for activation through several events, including phosphorylation. It is important to note that RAS-RAF binding is not enough to trigger RAF activation but that it increases the likelihood of other effector proteins’ activating RAF by recruiting them to the cell membrane. Moreover, the conformational change might play a vital role in identifying a docking site for the MEK substrate on RAF [20,50,51]. MEK1 and MEK2 are activated downstream of RAS and RAF via the phosphorylation of two serine residues that reside within the activation domain [52]. These signals trigger ERK, which amplifies them by phosphorylating nuclear and cytosolic targets. This amplified phosphorylated signaling leads to diverse and context-dependent biological outcomes [53].

The alternation of MEK and ERK actions will therefore have marked effects on cell proliferation and growth due to their crucial role as transcriptional factors [53]. ERK will be transferred to the nucleus, where it phosphorylates various transcription factors, including the cAMP response element-binding protein, ELK-1, FOS, GATA1, and others, which, through binding, enhance the activity of many genes, encoding several cytokines and growth factors, thereby impacting cell division and preventing hematopoietic cell death [54]. Dysregulated functioning of this pathway can lead to irregular cell proliferation, which in turn can cause various malfunctions such as drug resistance, leukemic transformation, abrogation of cytokine dependency, and autocrine cytokine secretion. It is imperative to maintain proper regulation of the system to prevent such aberrations [20,28,55].

### 2.4. Downstream Action of RAS Protein in the PI3K Pathway

The PI3K (phosphatidylinositol 3 kinase) pathway stands as the second most well recognized *RAS* effector pathway, playing a crucial role in controlling numerous cellular processes [56]. Moreover, the PI3K-AKT-mTOR pathway is undeniably abnormally upregulated in malignancies, particularly in AML, making it a significant target for cancer treatment [57]. Uncontrolled PI3K activation is present in 50% of AML cases [58,59]. Additionally, mTORC1 is widely reported to be a critical pathway in AML [56,60]. A recent study demonstrated a decline in mTORC1 signaling activity along with AML progression. On the other hand, at the time of maximal chemotherapy response, the mTORC1 signaling activity was found to be elevated and to have a positive correlation with a leukemia stemness transcriptional profile [61].

## 3. Pathological Sustained RAS Switch-On in AML

In acute myeloid leukemia (AML), mutations in the RAS gene family, particularly in NRAS and KRAS, are clinically significant. These mutations can drive oncogenesis by constitutively activating signaling pathways that lead to uncontrolled cell proliferation. Several point mutations in *RAS* proto-oncogene result in the sustained switching-on of RAS signaling through different mechanisms that hinder GTP hydrolysis [8,13,23,62]. The presence of point mutations in the *RAS* gene codons 12, 13, or 61 occurs specifically in tumor cells only but not in healthy cells [63]. Recent research has demonstrated that not all RAS mutations are the same, since different amino acid substitutions at a particular location can result in mutant RAS proteins with unique biochemical, structural, and signaling characteristics [8,9,13,23,33,62,64,65,66,67]. For instance, two hot spots of *RAS* mutations have been identified: the mutations at glycines 12 and 13 (G12/13), which lessen RAS association with Ras GAPs, and at glutamine 61 (Q61), which decrease the intrinsic GTPase activity of RAS [40,41,42,68].

The “two hits” theory posits that effective leukemogenesis requires multiple genetic alternations that deregulate several cellular programs [27,40,41,42,57,69]. For instance, transcription factors such as PML-RARA and MLL/AF9 are essential to halting myeloid lineage cellular differentiation, granting the second crucial event for leukemogenesis [70]. These, however, must be preceded by mutations in genes such *DNMT3A*, *NPM1*, *FLT3*, or *RAS*, which are essential to initiating leukemogenesis, all together contributing to disordered cellular proliferation and upregulation of anti-apoptotic genes, commonly found in AML patients [71]. There is a major interdependence between the two molecular hits as the alteration in the transcriptional factors’ control regulation can alter signal transduction effectors. On the other hand, mutations in signal transduction effectors alter the expression of various transcription factors that are essential for normal myeloid lineage differentiation [72,73,74].

Importantly, recent studies claim that *RAS* mutation may develop during leukemia clones’ evolution rather than being the original leukemogenic event [75,76]. Additional data indicate that, before overt leukemia manifests, patients with myelodysplasia have a significant rate of *RAS* mutation [77]. Moreover, the *RAS* gene may be overexpressed in leukemic cells without actual mutation in the gene itself due to mutations in its promotor [50,78]. Together, these findings represent a paradigm shift in our understanding of AML biology and open the new challenge of navigating diagnostic and therapeutic processes in AML patients with *RAS* mutations.

**Figure 3:**
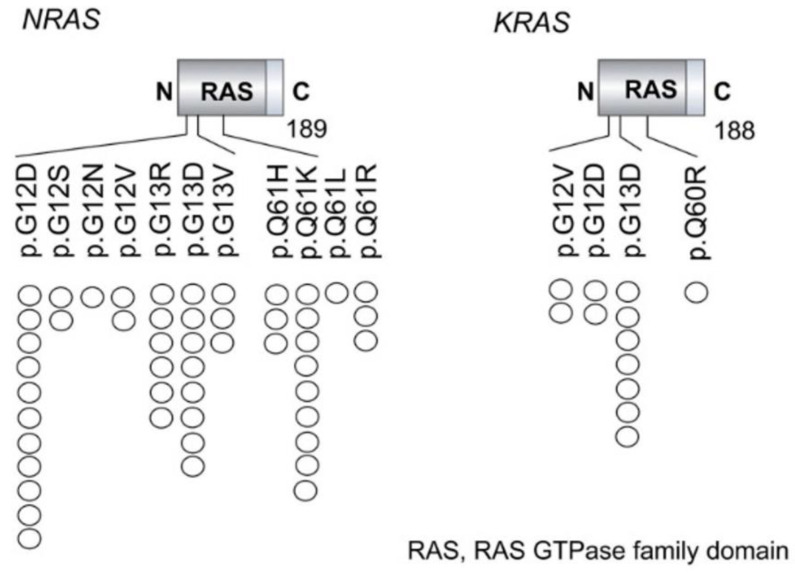
Schematic Representation of the Most Common Mutations Affecting KRAS and NRAS. This schematic illustrates the most common mutations in KRAS, NRAS, and RRAS2, highlighting the involvement of key functional gene domains (adapted from [79]).

The most common NRAS and KRAS mutations occurring in AML are summarized in Table 1 and are depicted in Figure 3.

## 4. RAS, FAB AML Subtypes and MDS

Several studies observed an association between FAB AML M4 subtype and *RAS* mutation [80,81,82]. In contrast, other studies have found no such association [21]. Astonishingly, *RAS* mutations are commonly observed in myelodysplastic syndrome (MDS) which progresses to secondary AML, suggesting their role in leukemic transformation from MDS to AML. In MDS, *RAS* mutations are correlated with more aggressive disease subtypes, higher IPSS-M risk, and reduced event-free survival (EFS) and overall survival (OS) [83]. A recent cohort demonstrated that patients have an increased risk of leukemic transformation, which is primarily associated with *NRAS* rather than *KRAS* mutation [84]. Moreover, one study revealed that transformed AML is the only subtype that was significantly more frequent among those with *RAS* mutation; this observation is related to the crucial effect of *RAS* mutations as a co-factor in AML transformation from MDS.

## 5. Cytogenetic and Molecular Landscape of RAS-Mutated AML

Acute myeloid leukemia can be categorized into three disease risk categories—favorable, moderate, and adverse—depending on the results of conventional cytogenetic analysis [85]. When it comes to cytogenetics and their relationship with *RAS* mutation status, one study revealed that *NRAS* mutation was significantly more common in the subgroup with t(3;5) translocation and less frequent in the t(15;17) subgroup [22]. AML cases with inv(16) (p13q22) were found to be significantly associated with *KRAS* mutation, as it was found that 23% of *KRAS* occurred in the inv(16) group of patients. On the other hand, no remarkable difference in the frequency of *NRAS* or *KRAS* mutation was observed between the different cytogenetic risk groups [22]. A different study revealed that patients with inv(16)/t(16;16) and inv(3)/t(3;3) showed a notably higher frequency of *NRAS* mutation compared with the t(15;17) group of patients [86,87]. Furthermore, the study found that *NRAS* mutation was under-represented in patients with −5 and/or −7 karyotype and in cohorts with complex karyotypes [86]. In a study of cytogenetically normal AML, *KRAS* mutation was extremely rare, and *NRAS* mutation was found only in 10% of the cases [88]. On the other hand, a multivariate analysis revealed that *KRAS* mutation, but not *NRAS* mutation, was an adverse independent prognostic factor in the cohort of patients with cytogenetically normal AML but not in the whole cohort of AML patients [89].

Core-binding factor (CBF)-AML patients have a high rate of *RAS* family mutations [62,87,90]. *NRAS* or *KRAS* mutation is more frequent in the inv(16) subgroup than in patients with t(8;21) [91]. It is still unclear what impact *RAS* mutations have on CBF-AML because there is no significant correlation between them and survival outcomes [91]. In a study of 215 bone marrow samples from CBF-AML by high-throughput sequencing, high *NRAS*:*KRAS* mutant allele ratios were associated with the absence of *KIT* or *FLT3* mutations and a favorable outcome [92]. However, a recent study performed on CBF-AML patients demonstrated that *RAS*-mutated cases demonstrated shorter overall survival rates (HR: 1.520; *p* = 0.04) compared with wild-type *RAS* [87]. Astonishingly, *RAS* mutations had no prognostic effect on overall survival or disease-free survival within various cytogenetic subgroups [22,86,87].

In addition to conventional cytogenetics, a common mutational status that defines AML mutations such as *CEBPA*, *NPM1*, and *FLT3*-ITD has been used extensively in clinical practice to determine prognoses and guide treatment approaches. Thanks to next-generation sequencing technologies, more recurrent somatic mutations in genes such as *TP53*, *IDH1*, *IDH2*, *RUNX1*, and *ASXL1* have been found to have prognostic value in AML and affect treatment decisions [93,94]. The most frequent concomitant mutations in *RAS*-mutated AML are *FLT3*, *NPM1*, *DNMT3A*, *ASXL1*, *IDH2*, and *RUNX1*; astonishingly, their incidence has not been shown to be significantly different from that in AML with wild-type *RAS* [2,95]. However, *RAS*-mutated AML has been shown to have significantly higher rates of concomitant *UA2F1*, *BCOR*, *EZH2*, and *BCORL1* mutations. Additionally, *TP53* was found to be significantly less commonly mutated in *RAS*-mutated AML than in wild-type *RAS* [2,25,95]. In contrast, one study found that the co-occurrence of *FLT3* and mutated *RAS* is rare [22]. Another report stated that there was no significant correlation between both *KRAS* and *NRAS* mutation status in AML and other mutations such as *CEBPA*, *FLT3*, *WT1*, *IDH1/2*, or *MLL*, but *KRAS* was associated with mutated *NPM1* [88]. In a contradictory report, *NRAS* was found to be mutated in approximately 30% of AML patients with a biallelic mutation of *CEPBA* and 20% of cases with a *NPM1* mutation [73,77,96,97]. Notably, aside from AML cases that present with mutant *RAS*, proteins that control RAS activation, such as PTPN11 and NF1, are also commonly mutated in AML.

## 6. RAS Mutation Prognosis in AML Patient Survival

Unfortunately, *RAS* mutations’ clinical impact is still not well understood, and research has produced contradictory results [8,24,98]. A few studies found that having a *RAS* mutation was significantly associated with a higher survival rate [63]. In contrast, one study stated that *RAS* mutations had no independent effect on AML prognosis [2,8,22,71,86]. Another two studies—the first featuring 232 AML patients with de novo AML, AML with myelodysplasia-related changes, or therapy-related AML at the time of induction chemotherapy who had next-generation sequencing performed prior to treatment, and the second featuring 239 newly diagnosed AML patients with various AML subtypes for whom *RAS* mutational status was determined using a next-generation sequencing myeloid panel—revealed that *KRAS* mutations were correlated with worse outcomes, while *NRAS* mutations had no impact on outcomes [8,23].

Of interest, the contribution of RAS mutations to AML evolution in different clinical settings might be slightly different. In pediatric AML, RAS mutations, primarily affecting NRAS, occur in approximately 15–20% of cases [2,8,71]. These mutations can be found as primary drivers or, more often, alongside other cytogenetic abnormalities, such as core-binding factor translocations. The interplay between RAS mutations and other genetic alterations is believed to influence the aggressiveness of the disease and its response to therapy. Some studies have suggested an association with a better initial response to induction therapy, while others indicate a risk of relapses. A meta-analysis evaluated the impact of *RAS* mutations on the overall survival of adult and pediatric patients with AML; surprisingly, the researchers found no significant prognostic effect of *RAS* mutations on adult patients’ survival but noted that *NRAS* mutations may be a vital prognostic marker in children with AML [14]. Furthermore, in a Japanese study conducted in a pediatric AML cohort, *NRAS* mutations were associated with a good outcome, while the impact of *KRAS* mutations could not be assessed due to the small number of patients [79]. In contrast, a German study on 372 pediatric AML patients showed that *KRAS* mutations were significantly associated with unsatisfactory therapeutic responses and increased early mortality rates; on the other hand, *NRAS* mutations were associated with better therapeutic responses [98]. Owing to the diverse pathophysiology of AML in pediatric cohorts compared to adults, pediatric AMLs display a unique mutational pattern in terms of upstream regulators of *RAS*. In particular, there is a marked reduction in *FLT3-ITD* mutations and a notable increase in *KIT* mutations in pediatric AMLs [99].

In summary, as mentioned, according to the clinical setting, RAS mutations may have slightly different roles and prognostic impacts. In adult AML, NRAS mutations are found in about 10–15% of cases, while KRAS mutations appear in 5–10% (Figure 2). These mutations frequently co-occur with FLT3-ITD, NPM1, and IDH mutations. The co-existence of RAS mutations with other genetic lesions contributes to the complexity of the disease and impacts therapeutic strategies. Particularly, RAS mutations can lead to clonal expansion and contribute to chemoresistance by enabling leukemic cells to survive under therapeutic pressure. They are often involved in relapse, as sub-clones harboring RAS mutations may become dominant following initial treatment success. Overall, this is usually translated into a poorer prognosis, although the impact varies depending on the mutational context and treatment regimen.

As far as myelodysplastic neoplasms (MDS) are concerned, RAS mutations are less common in early MDS but can emerge or become more prevalent as MDS progresses to AML, particularly in cases of secondary AML. In fact, these mutations contribute to the increased proliferative capacity and survival of leukemic cells, facilitating the transition from a pre-leukemic state to full-blown leukemia [77]. They may also interact with age-related changes in the bone marrow microenvironment (clonal hematopoiesis), exacerbating disease progression [13,100].

## 7. RAS Targeting Strategies

Despite major advances in the genetic characterization of AML, there is still a significant challenge in effectively applying our knowledge of common molecular abnormalities to develop safer and more potent treatments [101]. The development of chemical inhibitors for oncogenic “driver” proteins is based on two key principles. First, the affected protein must be critical for cancerous cell maintenance; second, blocking its action should have a favorable therapeutic index without causing any intolerable toxicity to healthy cells [102]. Both PI3K [103] and MAPK [50] play a crucial part in maintaining AML. Nevertheless, there is limited knowledge about the particular RAS effector pathways that are responsible for sustaining AML survival and growth. It is well known that directly targeting the RAS protein is difficult, as RAS lacks drug-binding pockets on its surface and has a high picomolar affinity for GTP, along with relatively high intracellular GTP concentrations [29]. On the other hand, the U.S. Food and Drug Administration’s approval of sotorasib (Lumakras, AMG510), which targets *KRAS*G12C mutation in non-small cell lung carcinoma patients, paved the road for searching for more opportunities to target *RAS* mutations in clinical settings [13,104].

Several druggable mechanisms have been proposed to target *RAS* directly or indirectly. These include targeting upstream RAS-activating molecules, developing chemical antibodies against the RAS protein itself, targeting downstream effecting molecules such as ERK and RAF (or combination therapy targeting both), RNA interference (RNAi) of *RAS* expression, and developing a drug that can target essential metabolic pathways associated with *RAS*, such as autophagy [13,69].

**Figure 4:**
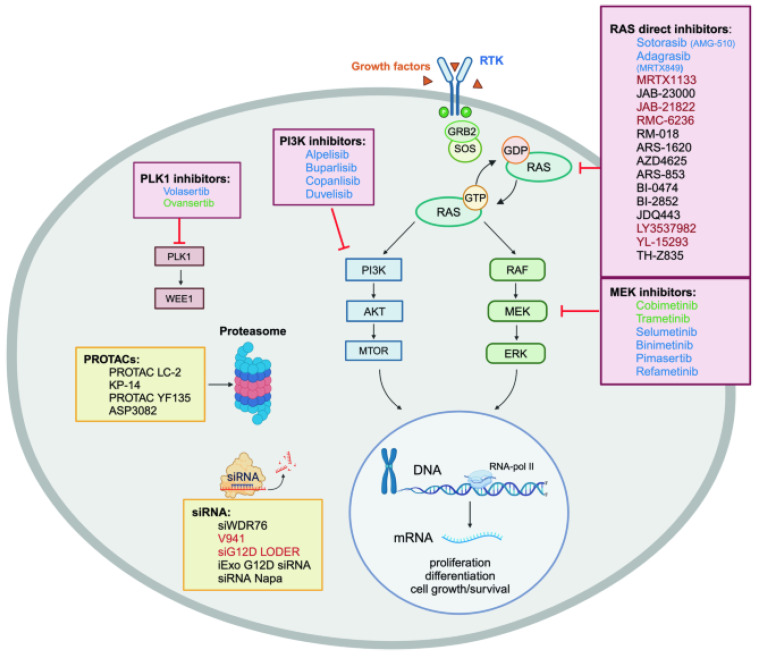
Novel and ongoing therapeutic strategies targeting RAS. This figure illustrates key therapeutic approaches for targeting RAS, focusing on direct RAS inhibitors, MEK inhibitors, PI3K inhibitors, and PLK1 inhibitors. The RAS signaling cascade is activated when growth factors bind to receptor tyrosine kinases (RTKs), leading to the exchange of GDP for GTP via GEFs like SOS. Active RAS then triggers downstream signaling through RAF and PI3K, regulating processes like cell proliferation and survival. Targets shown in green are in clinical trials for hematologic diseases, those in blue are FDA-approved for oncology, and preclinical/clinical drugs targeting RAS mutations in solid tumors are marked in black and red. Additional strategies include PROTACs and siRNA. Four essential therapeutic axes are emphasized: direct RAS inhibitors, MEK inhibitors, PI3K inhibitors, and PLK1 inhibitors (adapted from [13]).

Targeting RAS directly is an immensely challenging task [105]. To address this challenge, researchers tried to find some potential sites using computational methods [11,31,43,64]. For instance, inhibiting SOS–RAS interactions leads to trapping RAS in its inactive state and may carry therapeutic potential. Several small molecules that inhibit SOS–RAS interaction are under investigation [106]. In fact, the discovery of new sites on the surface of RAS and the creation of small-molecule inhibitors with high selectivity to prevent its interaction with activators and effectors is a promising development in the field of cancer treatment. These advancements could lead to the discovery of potent and innovative therapies for cancer [13,63]. In the following sections, we discuss some of the recently pursued preclinical and clinical strategies, further summarized in Table 2 and Figure 4.

### 7.1. Targeting the RAS Post-Translational Modifications

Post-translational modifications of proteins are vital for the regulation of physiological functions [107]. Protein farnesylation, a lipid post-translational modification, is one of the mechanisms that leads to cancer through its effect on proteins such as GTPase RAS [108]. Targeting the post-translational modifications of the RAS protein is one of the possible druggable methods that are undergoing extensive research. For instance, farnesyltransferase inhibitors, such as tipifarnib, showed astonishing results in preclinical studies; unfortunately, clinical results were unsatisfactory due to resistance driven by other prenylation pathways for RAS [109]. When farnesyltransferase is blocked, KRAS and NRAS sustain lipid modification through geranylgeranyl transferase, maintaining their physiologic function and hindering the drug activity [3,15,27,28,46,47,73]. Additionally, the combination of geranylgeranyl transferase inhibitors and farnesyltransferase inhibitors may have seemed promising, but research has revealed that it is not a viable option due to its high toxicity levels [3,20,28,46]. ICMT (isoprenylcysteine carboxylmethyltransferase) inhibitor compound 3, which inhibits RAS activity by blocking its post-translational methylation, showed a significant impairment of the membrane association of all four RAS isoforms, leading to downregulation of RAS downstream signaling pathways [110]. Additionally, it enhanced the survival of the in vivo model of *RAS*-mutated AML. A new ICMT inhibitor, UCM-1336, was shown to damage *RAS*-mutated AML cells in vitro and in vivo [111]. Additionally, targeting the palmitoylation/depalmitoylation cycle of post-translational modification is considered to be a promising druggable avenue for selectively halting the proliferation of hematologic malignancies with somatic *NRAS* mutations [112].

### 7.2. Targeting RAS Effector Pathways in AML

Many researchers have concentrated on blocking RAS effector pathways, since directly targeting RAS proteins has yielded unsatisfactory results. In the case of AML, inhibiting the two main oncogenic RAS effectors—the PI3K and MAPK pathways—has been found to be moderately effective. This may be due to the functional redundancy between these pathways and feedback loops that counteract the inhibition of these effector pathways [29]. Surprisingly, it was found that the RAS can stimulate the PI3K/AKT axis by itself or via the RAF/MEK/ERK pathway [20,28,54,73]. Additionally, many studies have shed light on the crucial role of PI3K/AKT axis activation in AML [47,58,73,113,114].

The *BRAF* gene, responsible for one of the three RAF proteins in humans, harbors a diverse range of somatic mutations, almost all of which are restricted to kinase domains [115]. It is a well-established fact that *BRAF* mutations in humans are consistently linked to melanoma [116]. On the other hand, these mutations are extremely rare in AML [117]. Recent research on AML cell lines has demonstrated the efficacy of pan-RAF inhibition through inducing apoptosis of the cells dependent on MCL1 for survival; as such, apoptosis downregulates MCL1 [78]. This research also demonstrated that the combination of a BCL2 inhibitor and pan-RAF inhibitor could overcome drug resistance to either compound alone in AML cell lines; furthermore, the combination induced long-term responses in ex vivo AML patient samples that were relapsed/refractory to azacitidine plus venetoclax. Another study reported that pan-RAF inhibitor LY3009120 can both accelerate apoptosis and hinder proliferation in *RAS*- or *FLT3*-mutated AML. Furthermore, LY3009120 combined with cytarabine showed a decrease in the chemotherapeutic resistance mediated by bone marrow-derived mesenchymal stem cells [50]. This combination also synergically potentiated the anti-apoptotic impact of sorafenib in patients harboring the *FLT3*-ITD mutation. Furthermore, the combination of low-dose cytarabine and LY3009120 significantly increased the anti-cancer effect on *RAS*-mutated AML cell lines [118]. Belvarafenib, a new type II pan-RAF kinase inhibitor, suppresses mutant monomeric BRAF proteins and activated RAF homo- and heterodimers [119]. Belvarafenib suppressed the growth of *RAS*-mutant human AML cell lines and *RAS*-mutant murine AMLs treated in vivo [120]. Additionally, belvarafenib and cobimetinib showed a synergistic effect in human AML cell lines and in three of five murine AMLs [120].

Despite the limited number of studies investigating MEK inhibitors in hematologic cancers, there is a preclinical rationale to target MAPK signaling in leukemias with *NRAS* or *NF1* mutations. These mutations have a crucial role in leukemogenesis, and targeting the MAPK pathway with MEK inhibitors may hold a modest therapeutic potential. For example, a phase II trial of selumetinib in AML patients with *NRAS* mutations showed a limited efficacy [18]. A phase 1/2 study conducted in patients with *RAS*-mutated myeloid neoplasms concluded that single-agent trametinib may have a therapeutic role [121]. One recent study investigated the effectiveness of trametinib combined with venetoclax in AML cell lines and demonstrated the therapeutic potential of the combination, given that hyperactivation of MAPK signaling is correlated with venetoclax resistance [122]. In contrast, a clinical trial found that the combination of azacitidine, venetoclax, and trametinib had only a humble effect in patients with relapsed/refractory AML, with more or less the same response rate of trametinib monotherapy being associated with a significant toxicity [123]. The combination of trametinib and pyrvinium pamoate significantly inhibited the proliferation of *RAS*-mutated primary AML cells ex vivo, especially in trametinib-resistant *PTPN11*- or *KRAS*-mutated samples [38]. Finally, an interesting study demonstrated the anti-leukemic effect of fentanyl in AML cell lines by suppressing the RAS/RAF/MEK/ERK and STAT5 pathways and independent from opioid receptors [124].

Small molecules targeting the PI3K/AKT/mTOR pathway have shown great potential in treating various human malignancies. The U.S. Food and Drug Administration has approved several small-molecule medications for various malignancies [125,126,127]. Despite substantial preclinical and clinical research on these molecules, both as monotherapies or in combination with traditional chemotherapeutic agents, they have not yet been effectively incorporated clinically for AML treatment. For example, Ragon et al. investigated buparlisib, a pan-PI3K inhibitor, in an open-label, non-randomized phase 1 trial [128]. Unfortunately, this biological inhibition did not improve the clinical response in AML patients, and buparlisib showed modest efficacy, with a median survival of only 75 days. Buparlisib induces p21-mediated G2/M cell cycle arrest and reduces the expression of NF-κB anti-apoptotic proteins [129].

The oral mTOR kinase inhibitor everolimus is used to prevent the rejection of transplanted organs and to treat several types of solid malignancies [130]. Everolimus was investigated in a randomized trial conducted in AML patients, but neither the cumulative incidence of recurrence nor overall survival was improved by it. Due to excessive mortality in the everolimus arm and insufficient evidence for effective disease management, the study involving randomized patients between consolidation chemotherapy cycles was halted [131]. Another mTOR kinase inhibitor, sirolimus, was tested in combination with MEC (mitoxantrone + etoposide + cytarabine) and found to be well tolerated in a clinical trial in patients with high-risk AML; however, no significant difference was observed in the objective response rates between patients with and without baseline mTORC1 activity [132].

Dual PI3K/mTOR inhibitors are a group of intriguing molecules that are currently under investigation. They can fully inhibit the abnormally activated PI3K/AKT/mTOR signaling pathway and block the compensatory activation of the AKT/mTOR pathway; this can improve patients’ outcomes. Gedatolisib is an extremely effective, selective, and ATP-competitive inhibitor of PI3Kα, PIK3γ, and mTOR [59]. Vargaftig et al. investigated gedatolisib for AML treatment in an open-label, prospective, single-arm, multicentric phase 2 clinical trial, but the trial was stopped as no objective response was achieved in any of the AML cohorts [133]. BEZ235 is a dual PI3K/mTOR inhibitor [58] that was evaluated in a phase 1 trial; however, there was no response in any of the 12 AML patients enrolled in this trial so far [134]. On the other hand, combining venetoclax with other therapeutic agents such as BEZ235 may offer a chance to overcome resistance to venetoclax [47,135].

ISC-4, a p-AKT inhibitor, was shown to induce apoptosis in leukemic stem cells and intensify the effectiveness of cytarabine [136]. Additionally, ISC-4 demonstrated a substantial survival benefit in animal models of AML. GSK2141795, another pan-AKT kinase inhibitor, repressed the proliferation of malignant cells in which the AKT pathway was activated in vitro and in vivo [137]. GSK2141795 was combined with trametinib (GSK1120212), a dual-specific MEK1 and MEK2 inhibitor, in a phase 2 clinical trial enrolling patients with RAS-mutated, relapsed/refractory AML; unfortunately, no patient achieved clinical remission, and the study was terminated due to the absence of any potential clinical efficacy [138].

In summary, several efforts to target RAS effector pathways in AML led to disappointing results, such as partial pathway deactivation, heterogeneous integral stimulation of the pathway, and drug-related toxicity. Therefore, drug combination strategies with acceptable toxicity profiles may pave the way for an effective treatment for AML [59].

#### Targeting RAL

RAL (RAS-like proteins), namely RALB and RALA, are activated in a similar way as RAS through RAL guanine exchange factors that enhance the exchange of GDP for GTP and are deactivated by RAL GTPase-activating proteins that promote their intrinsic GTPase activity [139,140]. RALB and RALA act as downstream targets of RAS, and, in both normal and neoplastic cellular states, they play important and unique roles in controlling vesicular trafficking, migration and invasion, cancer development, metastasis, and gene expression [140]. It was found that RALA was required for the anchorage-independent proliferation of malignant cells whereas RALB was essential for tumor survival but not for non-malignant cells, making RALB a promising therapeutic target [29,141]. Furthermore, it was reported that AML blasts express higher levels of RALB-TBK1 signaling compared to normal blood leukocytes, suggesting a pathophysiologic role for RALB signaling for AML development [141]. One preclinical study reported the efficacy of dinaciclib, a cyclin-dependent kinase (CDK) inhibitor, which showed RALB-dependent anti-leukemic activity in AML models—including patient-derived mouse xenografts—with trivial effects on non-malignant hematological progenitor cells [142]. Dinaciclib also acts independently by deactivation of CDK1, CDK2, CDK5, and CDK9. Thus, drugging RAL effector pathways is believed to be another promising therapeutic method of AML treatment. Dinaciclib can sensitize AML targets to natural killer cell-mediated cytotoxicity in human cell lines [143]. Furthermore, dinaciclib showed inhibition of AML cell growth through specific CDK inhibition and through its action on the ERK1/STAT3/MYC pathway [144].

**Table 2:** Selected RAS-targeting therapies.

*RAS* Targeting Strategy			
MOA	Drug Name	Current Stage	Registry Number/ Reference
Post-translational modifications			
Farnesyltransferase-i	Tipifarnib	CT phase 3	NCT00093990
ICMT	Compound 3	Preclinical	[110]
ICMT	UCM-1336	Preclinical	[28]
Targeting RAS effector pathways			
RAF-i	LY3009120	Preclinical	[118]
RAF-i	Belvarafenib	Preclinical	[120]
MEK-i	Selumetinib	CT phase 2	NCI200900250
MEK-i	Trametinib (+AZA/VEN)	CT phase 2	NCT04487106
Targeting PI3K/AKT/mTOR			
PI3K-i	Buparlisib	CT phase 1	NCT01396499
Dual PI3K/mTOR-i	Gedatolisib	CT phase 2	[133]
Dual PI3K/mTOR-i	BEZ235	CT phase 1	NCT01756118
p-AKT-i	GSK2141795	CT phase 2	[28]
mTOR-i	Everolimus		[131]
Targeting RAL			
RALB-dependent CDK1-i	Dinaciclib	Preclinical	[144]

MOA—Mechanism of action; CT—clinical trial; -i—inhibitor, ICMT—isoprenylcysteine carboxyl methyltransferase; AZA—azacitidine; VEN—venetoclax.

### 7.3. Synthetic Lethality

Synthetic lethality is a novel therapeutic approach being investigated to tackle resistance to anti-cancer drugs by using exploitable gene mutations. RNA interface and CRISPR-Cas9 are the fundamental technological approaches used for synthetic lethality screening [145]. Synthetic lethal gene suppression results in the death of only the tumor cells; the non-malignant cells are not affected [146]. Efforts to target *RAS* through synthetic lethality have been unsuccessful, prompting extensive research into alternate anti-cancer strategies directed against *KRAS*. *XPO1* inhibition and GATA2 transcription factor act as synthetic lethal players of *KRAS* and are being investigated using RNAi and CRISPR-Cas9 technology [147].

#### 7.3.1. RNA Interference

RNAi is considered a breakthrough discovery that paves the road for novel therapeutic targets that act through mRNA expression modulation. RNAi therapy targets the defective key genes involved in the pathological process rather than the mutant protein itself [148]. The primary challenge in realizing RNAi’s full therapeutic potential lies in the effective delivery of siRNA molecules, which act as RNAi’s therapeutic agents [148]. Several large-scale screens have used RNAi-mediated expression suppression to identify genes specifically required for *RAS*-mutated cells, but not for wild-type cells; however, those screens had minimal overlap between their results [38,147]. Nucleic acid therapeutics can potentiate the maturation of leukemic cells by directly enhancing the key genes responsible for maturation [3,149]. A recent report demonstrated the efficacy of lipopolymer/siFLT3 complexes as monotherapies and in combination with gilteritinib in FLT3-ITD AML animal models [150]. By using three-dimensional cell culture systems, mixed cell culture systems combining stromal cells, endothelial cells, immune cells, and malignant cells will lead to better development of RNAi [151].

#### 7.3.2. CRISPR/Cas9 Genome Engineering Libraries

siRNAs have not been fully accepted in clinical practice because of their low stability and drug delivery [152]. However, newer techniques for influencing gene expression, such as CRISPR/Cas9 genome engineering libraries, can be used as an alternative method for synthetic lethality [153]. CRISPR/Cas9 technologies use DNA rather than mRNA, resulting in gene knockout [154]. One group performed CRISPR-based screens on a panel of 14 human AML cell lines, revealing synthetic lethal interactions between the genes implicated in RAS handling, MAPK signaling, and mutated *RAS* in AML cell lines [155]. A recent study demonstrated that the *RUNX1-RUNX1T1* can be targeted and disrupted utilizing a dual intron-targeting CRISPR-Cas9-mediated strategy elucidating this novel methodology’s potential in future treatment of AML t(8;21) patients [156].

#### 7.3.3. Targeting RAS Through Microrna Modulation

MicroRNAs (miRNAs) are single-strand RNAs with a length of 19–22 nucleotides and are unequivocally responsible for controlling a wide range of post-transcriptional biological processes. The dysregulation of these mRNAs results in tumorigenesis. miRNAs can act as oncogenes or tumor suppressors [157]. The dysregulated expression of miRNAs leads to the halt of the physiologic hematopoietic process, resulting in the expansion of several hematological malignant clones. miRNAs carry the potential to be innovative in the diagnosis and treatment of AML [157]. Surprisingly, *RAS* can also be regulated by the level of certain miRNAs. The first insight into this concept was the discovery of Let7; Let7 is just the first of numerous human miRNAs that have been found to have the capacity to degrade *RAS* mRNA and reduce *RAS* expression [63].

Notably, leukemogenesis can be induced by aberrant miRNA expression through disruption of signaling networks. However, the expression of miRNAs can also be influenced by signaling networks [157]. For example, miR-24 expression is repressed in the subgroup of AML patients harboring t(8;21) through *RUNX1*. miR-24 downregulates MAPK signaling through its action on MKP7. MKP7 negatively regulates JNK kinases and mitogen-activated p38. On the other hand, upregulation of miR-24 promotes MAPK signaling by repressing MKP7, resulting in myeloid cell overgrowth [158,159]. Moreover, it was found that the PI3K/AKT/mTOR pathway can be repressed by miRNAs, resulting in promoting apoptosis and suppressing the proliferation of malignant myeloid cells [160]. One report stated that miR-181a can repress *KRAS*, *NRAS*, and *MAPK1* and attenuate AML growth directly through binding to the 3′-untranslated regions of *KRAS*, *NRAS*, and *MAPK1* [161]. Additionally, a recent study suggested that *EVI1* upregulated *NRAS* expression through epigenetic silencing of miR-124; thus, targeting miR-124 may have potential in regard to the treatment of AML with mutated *NRAS* [162]. Another study demonstrated that miR-133 upregulation can enhance doxorubicin sensitivity by repressing *EVI1* expression in leukemic cells [163].

## 8. RAS and Drug Resistance

Chemotherapy resistance is considered to be the key to treatment failure and the main cause of dismal prognosis and decreased overall survival in AML [164]. The high frequency of multidrug resistance significantly reduces the effectiveness of chemotherapy. Therefore, early assessment of chemoresistance is crucial to determining the optimal therapy in the current era of precision medicine. Unfortunately, *RAS* mutation is considered to be one of the most powerful underpinnings of resistance to various types of cancer treatments, even the newer targeted therapies in several types of malignancies—including AML [5,68,165,166].

Two types of drug resistance have been identified: primary and acquired [167]. Primary drug resistance denotes when the malignant cells remain in the non-proliferative G0 phase and exhibit an inherent insensitivity to treatment. This renders them impervious to the therapeutic effect of the medications from the start. Several mechanisms can result in primary drug resistance [168,169]; for example, variabilities in protein expression levels such as increased expression of ABCB1(P-gp) [170], epigenetic modifications [46], and somatic mutations. Secondary drug resistance may occur due to the presence of minor sub-clonal populations that can result in relapse. It is highly likely that these minuscule populations of cells can carry dominant drug-resistant sub-clonal mutations during the early stages of a disease, which may not be immediately detectable [171]. However, after the initial chemotherapy that specifically targets the overt mutations and eliminates the majority of cancerous cells, the sub-clonal cells multiply rapidly and take over the bulk of the tumor [39,172].

Cancer stem cell resistance is another rare, proposed mechanism that leads to drug resistance. It was first described in AML, and the concept has been applied to many other cancers [173]. Cancer stem cells have stemness characteristics as they are undifferentiated, have proliferative and self-renewal capabilities, and remain in a quiescent state, thereby resisting chemotherapy, which typically targets rapidly dividing cells. However, these cells have the proliferative capacity to maintain and re-expand after their initial elimination, resulting in relapse and resistance [174]. The RAS-dependent MAPK and PI3K/AKT pathways are known to be dysregulated in cancer stem cells [175]. *RAS*’s role in cancer stem cells is not fully clarified, but interactions with the Wnt/β-catenin pathway suggests involvement in cancer stem cell maintenance, plasticity, and increased survival [173].

One proposed mechanism by which *RAS* mutations are involved in chemotherapeutic resistance is through reactive oxygen species (ROS) generation/detoxification. The upregulated NRF2 pathway in malignant cells can effectively break down excessive ROS generated by chemotherapeutic agents, providing them with a shield against the treatment and ultimately leading to chemoresistance [176]. *KRAS* G12D mutations can upregulate *NRF2* transcription through stimulation of the TPA response element (TRE) via MAPK signaling, providing a protective advantage to the cancer cells against chemotherapy-induced ROS [165,177,178]. On the other hand, chemotherapeutic agents used in leukemic treatment may lead to RAS/RAF/MEK/ERK signaling activation and result in chemotherapeutic drug resistance. For example, doxorubicin is a topoisomerase II inhibitor commonly used in leukemia treatment [179]. Doxorubicin also acts through the generation of ROS by interacting with iron; ROS stops scavenging oxygen radicals from utilizing antioxidants to decrease the anti-apoptotic effect of doxorubicin [180]. Unfortunately, ROS induces the stimulation of RAS/RAF/MEK/ERK signaling, and PI3K/phosphatase activation upon oxidative stress can promote ERK1/2 activation [181].

*RAS* mutations have emerged as a probable resistance mechanism to treatment with *FLT3*, *IDH*, and *BCL2* inhibitors [2,166]. Furthermore, the compensatory activation of the PI3K-AKT and RAS–RAF-MAPK pathways have emerged as a plausible route of resistance in AML treated with TKIs such as inhibitors of *FLT3*, *BCL2*, and *IDH* [39]. It was reported that AML patients harboring *NRAS* mutations at the time of diagnosis sometimes acquired *FLT3* mutation at the time of disease relapse (and vice versa) [39,50,182,183]. The same authors reported an association between *NRAS* mutations and acquired resistance to *FLT3* kinase inhibitors in vitro. Additionally, by using targeted next-generation sequencing in patients with *FLT3*-mutated AML at the time of progression during treatment with FLT3-inhibitor gilteritinib, several mutations that activate RAS/MAPK pathway signaling have been identified, most commonly in *NRAS* or *KRAS*. These data elucidate a clonal selection mechanism of resistance [39]. *RAS* enrichment finally was found in one of transcriptionally distinct clusters of patients who developed resistance to BCL2-inhibitor Venetoclax [166]. This RAS-enriched cluster was characterized by sensitivity to mTOR and CDK inhibition and translated to specific therapeutic vulnerability. The presence of RAS in this bcl-2 inhibitor-resistant cohort caused sensitivity to several PI3K-AKT-mTOR, RAF, MEK, and ERK inhibitors, which, in consequence, led to sensitivity to inhibition of Hsp (heat shock protein) 90, (histone deacetylase) HDAC, (cyclin-dependent kinase) CDK and BRD (bromodomain protein)4 [166]. The crosstalk and signaling through alternative pathways are considered a crucial mechanism of resistance to RAS-targeted therapy and also in *RAS*-mutated malignancies. Therefore, characterization of the activation status of proteins downstream from RAS and PI3K in AML samples from patients with mutated *RAS* versus wild-type *RAS* could, when paralleled by transcriptomic/metabolomic profiling, serve as an Achilles heel when targeting mechanisms of drug resistance [39,79,86,184]. Furthermore, simultaneous administration of RAS-targeting therapeutic agents may have a great clinical impact either in combination with IDH, FLT3, or CDK inhibitors to prevent primary resistance in patients who have baseline *RAS* mutations or after treatment with IDH, BCL2, or FLT3 inhibitors in patients whose *RAS* mutations were detected during such therapy [137,166].

Strategies aimed at targeting RAS pathways are summarized in Figure 4.

## 9. Future Directions and Insights

Despite extensive research on RAS in acute myeloid leukemia (AML) and various cancers, significant gaps remain in understanding the role of RAS in disease progression and therapeutic resistance. The precise pathogenic roles of mutated KRAS and NRAS in AML have not yet been fully elucidated, primarily because RAS mutations typically occur as secondary events that contribute to the clonal evolution of AML. Co-existing genetic backgrounds may further influence the effects of these mutations. Comprehensive studies utilizing next-generation sequencing (NGS) at the single-cell level will be essential to resolving these complexities. The routine application of NGS in AML diagnostics will facilitate the collection of sufficient cases for detailed analyses, potentially clarifying the prognostic impact of RAS mutations, which appears to vary across different contexts—from negative to neutral to paradoxically favorable.

Targeting RAS in AML presents both challenges and opportunities. The efficacy of direct RAS inhibition is likely influenced by the tumor’s genetic background. For instance, RAS inhibition may be insufficient in cases where mTOR is activated by other oncogenes; conversely, it may show synergy with downstream therapies. Therefore, further research and drug discovery initiatives should prioritize the development of targeted inhibitors and the application of combination therapies. The development of specific RAS inhibitors—small molecules that directly target the RAS protein or its downstream effectors (e.g., MEK and ERK inhibitors)—should be prioritized, as they currently represent the most feasible pathway toward clinical applicability. Combining RAS inhibitors with existing therapies such as BCL2 inhibitors (e.g., venetoclax), which are currently being tested preclinically and pursued in phase I clinical trials, may enhance overall efficacy and overcome resistance; however, the results of these clinical efficacy studies are still pending. Future studies should also explore combination therapies targeting RAS alongside other dysregulated pathways, such as the PI3K-AKT-mTOR and JAK-STAT signaling pathways. This multi-targeted approach could disrupt compensatory survival signals, maximizing therapeutic impact.

Given that RAS mutations typically arise later in leukemia development and are enriched in relapse/refractory patients, further studies should focus on biomarker development. Identifying biomarkers that predict RAS pathway activation in AML patients could inform treatment strategies and improve the timely management of patients harboring RAS mutations. Profiling RAS mutations, post-translational modifications, and downstream pathway activity may also enhance patient selection for RAS-targeted therapies and improve therapeutic triage.

With the increasing successes of RAS targeting in solid tumors, the implementation of RAS-targeting strategies in clinical trials for AML is urgently needed. Designing clinical trials that assess the efficacy of RAS inhibitors or combination therapies specifically in RAS-mutant AML patients is crucial. Patient stratification based on increasingly available and cost-effective genetic profiling will enable tailored interventions.

Further preclinical research is necessary to characterize and understand resistance mechanisms. Investigating the mechanisms underlying resistance to RAS inhibition is vital; research should focus on how AML cells adapt to circumvent RAS-targeted therapies, informing refined therapeutic strategies. Additionally, a deeper understanding of how the bone marrow microenvironment influences RAS signaling in AML may yield novel therapeutic targets and enhance the effectiveness of RAS inhibitors. This knowledge could further leverage immunotherapeutic technologies such as CAR-T, CAR-NK, or CAR-M. Given the critical role of both cellular and non-cellular compartments in the development of diverse resistance mechanisms, exploring how extracellular factors, including cytokines and growth factors, influence RAS signaling could uncover additional therapeutic targets and improve the effectiveness of RAS inhibition.

Finally, educating patients—the ultimate beneficiaries of advancements in preclinical and clinical research—about the significance of genetic testing for RAS mutations is essential. This knowledge should inform personalized treatment options and enhance clinical decision-making.

## 10. Conclusions

The frequent occurrence of RAS mutations in AML plays a critical role in disease relapse and drug resistance through several mechanisms. Therefore, targeting RAS mutations is essential for prolonging overall survival and overcoming drug resistance in AML patients. Although finding effective ways to target mutant RAS has proven challenging, additional research into and exploration of various strategies, as suggested above, are necessary. Combining multiple inhibitors may enhance the blockade of mutant RAS activity, overcoming the resistance that often develops against single-target inhibitors. Such approaches could improve the therapeutic potential of induction, consolidation, and maintenance therapies for AML patients, ultimately leading to better patient outcomes.

## Figures and Tables

**Table 1:** Summary of the most relevant RAS mutations in AML.

Gene	Mutation	Molecular Features	Clinical Significance
*NRAS*	Codon 12 (G12):	G12D, G12A, G12V: These mutations result in the substitution of glycine, leading to impaired GTPase activity and the constitutive activation of NRAS.	Associated with poor prognosis and resistance to certain chemotherapies.
	Codon 13 (G13):	G13D: Similarly to G12 mutations, these alter the GTPase activity, promoting active signaling.	Often found in conjunction with other mutations, contributing to a complex mutational landscape.
	Codon 61 (Q61):	Q61L, Q61R: These mutations stabilize the GTP-bound form of NRAS, leading to persistent activation.	Frequently associated with aggressive disease phenotypes
*KRAS*	Codon 12 (G12):	G12D, G12V, G12C: Similarly to NRAS mutations, these disrupt the GTPase function, maintaining KRAS in an active state.	Contribute to treatment resistance and are often associated with a poor response to standard therapies.
	Codon 13 (G13):	G13D: Leads to constitutive activation and persistent downstream signaling.	Often associated with a distinct set of co-occurring mutations and can influence therapeutic outcomes.

## Data Availability

No new data were created or analyzed in this study. Data sharing is not applicable to this article.
